# Vegetation succession influences soil carbon sequestration in coastal alkali-saline soils in southeast China

**DOI:** 10.1038/s41598-018-28054-0

**Published:** 2018-06-27

**Authors:** Niu Li, Tianyun Shao, Tingshuo Zhu, Xiaohua Long, Xiumei Gao, Zhaopu Liu, Hongbo Shao, Zed Rengel

**Affiliations:** 1 College of Resources and Environmental Sciences, Nanjing Agricultural University, Nanjing, 210095 China; 2 Salt-soil Agricultural Center, Institute of Agricultural Resources and Environment, Jiangsu Academy of Agricultural Sciences, Zhongling Street 50, Nanjing, 210014 China; 3 Soil Science and Plant Nutrition, School of Earth and Environment, The University of Western Australia, 35 Stirling Highway, Crawley, WA 6009 Australia

## Abstract

The area of saline soils accounts for 8% of the earth’s surface, making these soils an important terrestrial carbon sink. Soil organic carbon (SOC), microbial biomass carbon (MBC), dissolved organic carbon (DOC), soil enzyme activity, and soil bacterial abundance and biodiversity were measured in four successive coastal tidal flat ecosystems representing: bare saline soil (BS), *Suaeda glauca* land (SL), *Imperata cylindrica* grassland (IG), and *Jerusalem artichoke* field (JF). A decrease in soil salt content resulted in increased SOC content. With vegetation succession, MBC and DOC concentrations showed a positive trend, and activities of soil urease, catalase, invertase and alkaline phosphatase increased. A next-generation, Illumina-based sequencing approach showed that *Proteobacteria, Acidobacteria, Chloroflexi, Bacteroidetes, Gemmatimonadetes, Actinobacteria, Nitrospirae* and *Planctomycetes* were the dominant bacterial communities (a total of 597 taxa were detected, and 27 genera showed significant differences among the vegetation communities). Bacterial diversity at two soil depths was enhanced with the succession of vegetation ecosystems, with the increases in operational taxonomic units (OTUs) and the Shannon and Chao1 indices ranked in the order: JF > IG > SL > BS. The SOC and C/N were the most determinant factors influencing diversity of bacterial communities in the succession ecosystems.

## Introduction

The acceleration of greenhouse gas emissions is one of the primary concerns in the twenty-first century^1^. An increase in atmospheric temperatures and corresponding climate change and variability are attributed to the increasing levels of atmospheric carbon dioxide (CO_2_) and other greenhouse gases. The concentration of CO_2_ has increased by 31% from 280 μL/L in 1850 to 380 μL/L in 2004. The Earth surface temperature has increased between 0.4 and 0.8 °C (0.6 ± 0.2 °C) since the late 19^th^ century^2^. An estimate of the expected rise in average surface air temperature globally is between 1 and 3.5 °C by year 2100^3^. Carbon sequestration in soil reduces the rate of enrichment of atmospheric CO_2_ concentration, which can contribute to mitigating climate change^4^.

Soil salinization is an important process, affecting about 8.31 × 10^8^ ha of soil resources worldwide^5^. The total area of saline soil in China is about 3.6 × 10^7^ ha, accounting for 4.9% percent of total available land in the country^6^. The studies on sequestering atmospheric carbon in China have been focused mainly on forest, grassland and farmland soils^7^, with little knowledge on soil carbon sequestration in coastal alkali-saline soils.

Soil contains the largest pool of terrestrial organic carbon in the biosphere, storing more carbon than plants and atmosphere combined^8^, with 73% of soil C contained in soil organic matter^9^. It is estimated that the soil carbon reserve is about 2.5~3.0 times the vegetation carbon reserve in terrestrial ecosystems, and 2~3 times the carbon reserve in the atmospheric carbon pool. Therefore, relatively small variations in the C reserves in the soil organic C pool may influence substantially the concentration of CO_2_ in the atmosphere^10^.

Some studies indicated that vegetation succession can improve soil carbon sequestration capacity^11^. In addition, many studies on soil carbon dynamics agreed that the storage capacity and relative distribution of soil carbon are related to the vegetation type^12^. The reason is mainly due to different plant forms influencing soil physical and chemical properties, litter chemistry, detritus input and rooting depth^13^. However, such knowledge on the relationships between soil carbon and soil biotic and abiotic characteristics in different vegetation types in coastal alkali-saline soils is lacking.

Jiangsu Province (China) has abundant tidal flats with a total area of 6.0 × 10^5^ hectares, representing a quarter of the total area of tidal flats in the country^14^. The coastal tidal flats in Dafeng area are an important part of tidal flats in Jiangsu Province, representing the largest tidal wetland in eastern Asia. Moreover, tidal flats in this area still expand at the rate of 50~200 m per year due to a large amount of sediment deposition by Yellow River and Yangtze River^14,15^. The experimental areas were undisturbed and maintain the original natural state.

The hydromorphic soils are easily affected by seawater intrusion and groundwater recharge and are vulnerable to becoming saline. Halophytic vegetation forms on saline soils, with salt-tolerant *Suaeda glauca* being a pioneer plant species^16^. Its residues cover soil surface, increase soil organic matter, improve soil fertility and alleviate the effects of salt accumulation on the soil surface. With a gradual decrease in soil salinity, communities of herbaceous perennials (e.g. *Imperata cylindrica*) appear in succession and begin to dominate^17^. In such circumstances, soil salinity gradually decreases, and cultivation of salt-tolerant *Helianthus tuberosum* (Jerusalem artichoke) becomes possible as the advanced stage in the process of vegetation succession. However, there is little knowledge about soil carbon sequestration along the sequence of vegetation succession in the tidal flats.

Soil microbial community plays a key role in mediating ecosystem C and N cycling^18^. However, plant community structure, soil pH, moisture, organic carbon, temperature, and climatic and environmental factors may affect soil microbial community composition and biomass^19^. Specific soil bacterial assemblages were responsible for decomposition of individual soil organic compounds. However, there is little knowledge about soil microbial communities in different native vegetation types in coastal saline alkali soils, particularly regarding the effects of specific microbial communities on soil carbon sequestration.

This study was aimed at determining soil physicochemical and biological properties along a vegetation succession in the coastal tidal flats in Dafeng area, China. In particular, we characterized changes in SOC, MBC, DOC, soil enzyme activities and soil bacterial diversity as influenced by the process of vegetation succession.

## Materials and Methods

### Study area

The studied area is located at Dafeng Nature Reserve of Jiangsu Province (33°00.688′N, 120°50.755′E) with an area of 78,000 hectares, of which core area is 2,668 hectares, buffer zone is 2,220 hectares, and experimental area is 73,112 hectares. The nature reserve faces the Yellow Sea and became land about 50 years ago. It is a typical coastal wetland. The main wetland types include tidal flats, seasonal rivers, and partially constructed wetlands. There are a large number of woodlands, reeds, and bare land, which provide habitat for a large number of animals and plants. The area has the typical monsoon climate transitioning from the warm-temperate zone to the north subtropical zone, with 2,280 h of average annual sunshine, 14.4–15.5 °C average annual temperature, and 214 frost-free days. The annual precipitation ranges from 785–1310 mm (most occurring from late June to August). In this research, the spatio-temporal substitution method was used to monitor changes in plants and soils occurring along a vegetative chronosequence^20^. The typical vegetations in this area for characterizing succession were Jerusalem artichoke field (JF), *Imperata cylindrica* grassland (IG), *Suaeda glauca* land (SL) and bare saline alkali soil (BS) chosen as control. The BS, SL and IG areas were undisturbed. Jerusalem artichoke was planted with inter-row (60 cm) and intra-row distance between plants (40 cm) on the natural saline-alkaline land 8 years ago after mowing the grass meadows; there was no fertilizer and no irrigation used ever. Jerusalem artichoke tubers were never collected; instead, tubers left in the ground germinated and grew unaffected by humans. Three representative sample plots 5 × 5 m were selected as replicates in each vegetation type.

### Sample collection and pretreatment

In April of 2014, soil samples were taken randomly at five points in an “S” pattern in each replicate plot using a soil auger (6-cm diameter) at two depths (0–10 and 10–20 cm). Soil samples were packed in individual sterile plastic bags, and transferred on ice to the laboratory. Soil samples were air-dried, passed through a 2-mm sieve, and stored at room temperature for measuring physicochemical properties (EC, pH, total N, total P, SOC). Soil bulk density was measured using the separately collected soil cores (100 cm^3^) by the volumetric ring method.

In July of 2014, soil samples were collected as described above and placed into DNA-free polythene bags. The samples were kept on dry ice for transport to the laboratory, and were then stored at −20 °C for subsequent DNA extraction, microbial biomass carbon (MBC) and dissolved organic carbon (DOC) analyses. The remaining part of each soil sample was air-dried, passed through a 2-mm sieve, and stored at room temperature for soil organic carbon (SOC), soil enzyme activity, total N, and total P analyses.

### Analytical methods

#### Soil physicochemical properties

Electrical conductivity (EC) and soil pH were measured in 1:5 (soil:water) suspension after end-over-end shaking for 15 minutes at 25 °C. Total nitrogen (TN) was analyzed by Kjeldahl method^21^. Total phosphorus (TP) was extracted with HF-HNO_3_-HClO_4_ and then determined by molybdenum antimony blue colorimetry^22^.

#### Soil organ carbon (SOC), microbial biomass carbon (MBC) and dissolved organic carbon (DOC)

SOC was measured by dichromate oxidation^23^. Carbon (C) from the microbial biomass was extracted using the chloroform fumigation and extraction method^24^. For this, 12.5 g of soil (fresh weight basis) were subjected to 48-h fumigation with chloroform in a glass desiccator. Triplicate subsamples of fumigated and non-fumigated soils from each of the three field soil samples were shaken for 30 min at 200 strokes per minute with 0.5 M K_2_SO_4_ at a ratio of 5:1 (extractant to fresh soil weight) and filtered through medium-speed quantitative filter paper, then placed at −15 °C until measurements. Organic carbon concentration in the filtrate was measured by a Shimadzu TOC-5000A analyzer (Shimadzu Corp., Kyoto, Japan). MBC was calculated as the difference between fumigated and non-fumigated samples. DOC concentration in soil samples was determined by a TOC analyzer (Elementar Analysensysteme GmbH, Germany)^25^.

#### Soil enzymes

Urease activity (URE) was determined by measuring released NH_4_^+^-N from the soil amended with urea^26^. Catalase activity (CAT) determination was based on the volume of KMnO_4_ necessary for titration of unused H_2_O_2_^27^. Alkaline phosphatase (ALP) activity was measured within 24 h of sampling according to the published method^28^. Invertase activity was assessed using colorimetric determination of reducing sugars that react with 3,5-dinitrosalicylic acid upon incubation of soil in buffered (0.17 M modified universal buffer, pH 5.5) sucrose solution and toluene at 37 °C for 24 h^29^.

#### DNA extraction

Soil DNA extraction was performed on three samples randomly collected in each field site in July. Total soil DNA was extracted from 0.35 g of soil (after it had passed through a 1-mm sieve) using the PowerSoil DNA Isolation Kit (MO BIO Laboratories Inc., Carlsbad, CA, USA) according to the instructions provided by the manufacturer. Genomic DNA concentration and purity were determined by NanoDrop spectrophotometry (Thermo Scientific, Wilmington, DE, USA). DNA samples were stored at −80 °C^30^.

#### PCR amplification of bacterial 16S rRNA genes

PCR amplification of the V3-V4 region of 16S rDNA was conducted using the universal primers, 577 F (5′-AYTGGGYDTAAAGNG-3′) and 926 R (5′-CCGTCAATTCMTTTRAGT-3′). Amplification reactions were performed in 25-μL volume containing 12.5 μL Premix Ex TaqTM Hot Start Version (Takara Biotechnology Co. Ltd, Dalian, China), 0.1 μM of each primer, and 20 ng of template. PCR was conducted under the following conditions: 98 °C for 3 s, 35 cycles of denaturation at 98 °C for 10 s, annealing at 54 °C for 30 s, extension at 72 °C for 45 s, and a final extension at 72 °C for 10 min, followed by cooling to 4 °C. Amplicon pyrosequencing was performed on an Illumina MiSeq platform at LC-Bio Technology Co., Ltd, Hangzhou, Zhejiang Province, China.

#### Illumina MiSeq sequencing of 16S rRNA genes

QIIME (Quantitative Insights Into Microbial Ecology) quality filters were used for filtering the reads. The CD-HIT pipeline was used to pick operational taxonomic units (OTUs) and make OTU table. The sequences were assigned to OTUs with similarity of 97%. The representative sequences were chosen for each OTU, and RDP (Ribosomal Database Project) classifier was used to assign taxonomic data to each representative sequence^31^. In order to estimate Alpha Diversity, the OTU table was rarified, and four metrics were calculated: Chao 1 index to estimate the richness, the Observed OTUs metric as the count of unique OTUs found in the sample, and Shannon and Simpson indices to estimate diversity^32^.

#### Statistical analysis

Soil samples in each vegetation ecosystem and all measurements were replicated thrice. The mean values of all parameters were taken from the three field replicates, and the standard error of the means was calculated. Statistical analyses were performed using Microsoft Excel 2010 and SPSS Statistics19.0 (IBM, Armonk, New York, USA). One-way Analysis of Variance (ANOVA) was performed to compare the mean values for the different field sites. Correlation analyses were done using the Pearson correlation method with significance defined at the 0.05 level unless otherwise stated. Individual means were compared using the least significant difference test at 5% significance level. Redundancy discrimination analysis (RDA) was used to detect the bacterial community distribution in relation to environmental explanatory variables using Vegan 2.3.0, a package of R functions for community ecology.

## Results

### Soil physicochemical properties

Soil properties at each site were presented in Table 1. There was a decline in soil pH from the bare soil to *Suaeda glauca* land and Jerusalem artichoke field at both soil depths. EC in both soil layers decreased with the succession of vegetation: BS > SL > IG > JF.

**Table 1:** Soil physicochemical properties (sample collection in April) at four sites in Dafeng coastal saline alkali land.

Sites	Soil layer	pH	EC (µS·cm^−1^)	TN (g·kg^−1^)	SOC (g·kg^−1^)	Soil bulk density (g·cm^−3^)
BS	0–10 cm	8.53 ± 0.85a	632 ± 28a	0.43 ± 0.01d	6.4 ± 0.27b	1.63 ± 0.01a
	10–20 cm	8.35 ± 0.19ab	329 ± 20a	0.37 ± 0.04c	4.8 ± 0.06c	1.97 ± 0.02a
SL	0–10 cm	8.19 ± 0.40b	270 ± 12b	0.81 ± 0.04c	12.0 ± 0.55a	1.39 ± 0.02b
	10–20 cm	8.17 ± 0.40b	232 ± 8b	0.52 ± 0.02b	7.9 ± 0.41b	1.59 ± 0.02ab
IG	0–10 cm	8.78 ± 0.15a	116 ± 8c	1.00 ± 0.02b	12.3 ± 0.31a	1.32 ± 0.04bc
	10–20 cm	8.74 ± 0.15a	79 ± 6c	0.76 ± 0.02a	10.1 ± 0.42ab	1.56 ± 0.02ab
JF	0–10 cm	7.74 ± 0.48c	79 ± 5c	1.15 ± 0.05a	13.1 ± 0.56a	1.29 ± 0.03c
	10–20 cm	7.41 ± 0.03c	60 ± 5c	0.77 ± 0.01a	12.1 ± 1.42a	1.34 ± 0.01b

Different letters in a column denote significant difference among the four sites (P ≤ 0.05) separately for the two depths. Means ± standard error (n = 3).

The content of TN as well as SOC in both soil layers increased along the vegetation sequence from BS to JF (Table 1). In contrast, soil bulk density at both soil depths decreased in the order: BS > SL > IG > JF.

### Soil organic carbon (SOC), microbial biomass carbon (MBC) and dissolved organic carbon (DOC)

Dynamics of SOC content under different vegetation types in July was presented in Table 2. As expected, SOC increased along the vegetation sequence from BS to JF at both soil depths.

**Table 2:** SOC content of different vegetation types in July (g·kg^−1^).

Different vegetation types	SOC (g·kg^−1^)	
	0–10 cm	10–20 cm
BS	5.3 ± 0.3c	4.3 ± 0.2d
SL	11 ± 0.2b	7.4 ± 0.1c
IG	11.5 ± 0.7b	8.9 ± 0.3b
JF	12.3 ± 0.8a	11.0 ± 0.3a

Different letters in a single column denote significant difference (P ≤ 0.05). Means of three replicates ± standard error.

The mean values of MBC and DOC are presented in Table 3. At 0–10 cm soil depth, JF soil showed the highest MBC, followed by IG, SL and BS. A similar ranking was also obtained at 10–20 cm soil depth, except there was no difference between SL and BS. Regarding DOC, JF and IG showed higher values than SL and BS at both soil depths. The DOC content was higher in IG than JF at 0–10 cm soil depth, but reverse was true at 10–20 cm depth.

**Table 3:** Soil MBC and DOC content under different vegetation types.

Different vegetation types	MBC (mg· kg^−1^)		DOC (mg· kg^−1^)	
	0–10 cm	10–20 cm	0–10 cm	10–20 cm
BS	25 ± 1.2d	24 ± 1.9c	13 ± 1.9d	11 ± 2.0d
SL	45 ± 1.9c	25 ± 2.1c	23 ± 1.7c	19 ± 2.6c
IG	130 ± 2.8b	37 ± 1.7b	43 ± 2.8a	27 ± 1.2b
JF	431 ± 9.2a	179 ± 1.1a	31 ± 2.6b	38 ± 0.9a

Different letters in a single column indicate significant (P ≤ 0.05) differences. Means ± standard error (n = 3).

### Soil enzyme activity

**Figure 1:**
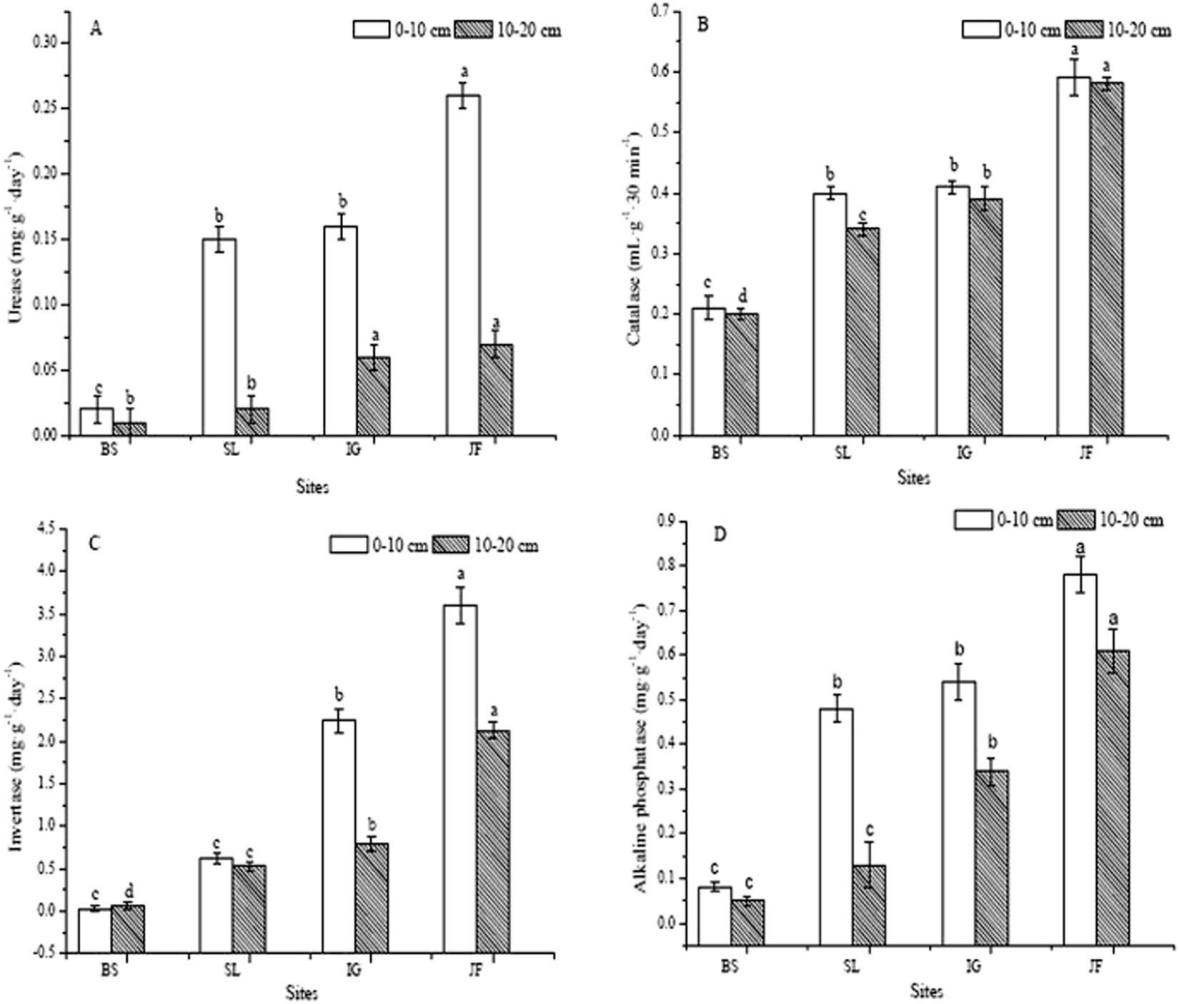
The urease (**A**), catalase (**B**), invertase (**C**) and alkaline phosphatase (**D**) activities in soil at four different sites along vegetation succession in Dafeng coastal saline alkali land. The scale of the Y-axis differs in different graphs. Different letters in each graph at each soil depth denote significant differences among sites (P ≤ 0.05).

Soil enzyme activities tended to increase along vegetation succession (Fig. 1). At 10–20 cm depth, all four enzymes showed higher activities in JF and IG soils than SI and BS. A similar situation was recorded for the 0–10 cm depth as well, except that no difference between IG and SL was noted for urease and alkaline phosphatase activities.

Alkaline phosphatase activity was correlated with urease (0.58, p ≤ 0.05), and catalase activity was correlated with invertase (0.79, p < 0.01) (Table 4). The SOC content had a significant positive correlation with urease (0.64, p < 0.01) and invertase activity (0.89, p < 0.01). In contrast, soil EC had a significant negative correlation with urease (−0.87, p < 0.01), alkaline phosphatase (−0.66, p < 0.01) and invertase activity (−0.82, p < 0.01).

**Table 4:** Simple correlation coefficients between soil physicochemical properties and enzyme activity.

	Urease	Catalase	Alkaline phosphatase	Invertase	pH	Soil bulk density	SOC	TN	TP
Catalase	0.08								
Alkaline phosphatase	0.58**	−0.04							
Invertase	0.56	0.79**	0.41						
pH	−0.02	−0.07	−0.01	−0.02					
Soil bulk density	−0.82**	−0.32	−0.42	−0.74**	0.43				
SOC	0.64*	0.50	0.55	0.89**	−0.56	−0.89**			
TN	0.87**	−0.12	0.39	0.29	0.33	−0.55	0.28		
TP	0.34	0.84**	0.19	0.85**	−0.40	−0.68**	0.80**	0.02	
EC	−0.87**	−0.39	−0.66**	−0.82**	0.33	−0.93**	−0.91**	−0.57	−0.69

**Significant at (p < 0.01); *significant at (p ≤ 0.05). The significant values were set in bold font.

### Soil bacterial diversity and richness indices

We obtained a total of 9,831,155 valid reads through a sequence optimization process. A median sequence length of each read was 16.77 M based on the quality filtering. More than 10,000 reads were selected randomly from each sample to determine rarefaction curves, richness and diversity. Rarefaction curves determine the extent of species diversity through a slope. A total of 367,316 OTUs were identified at a 97% similarity cutoff point.

**Figure 2:**
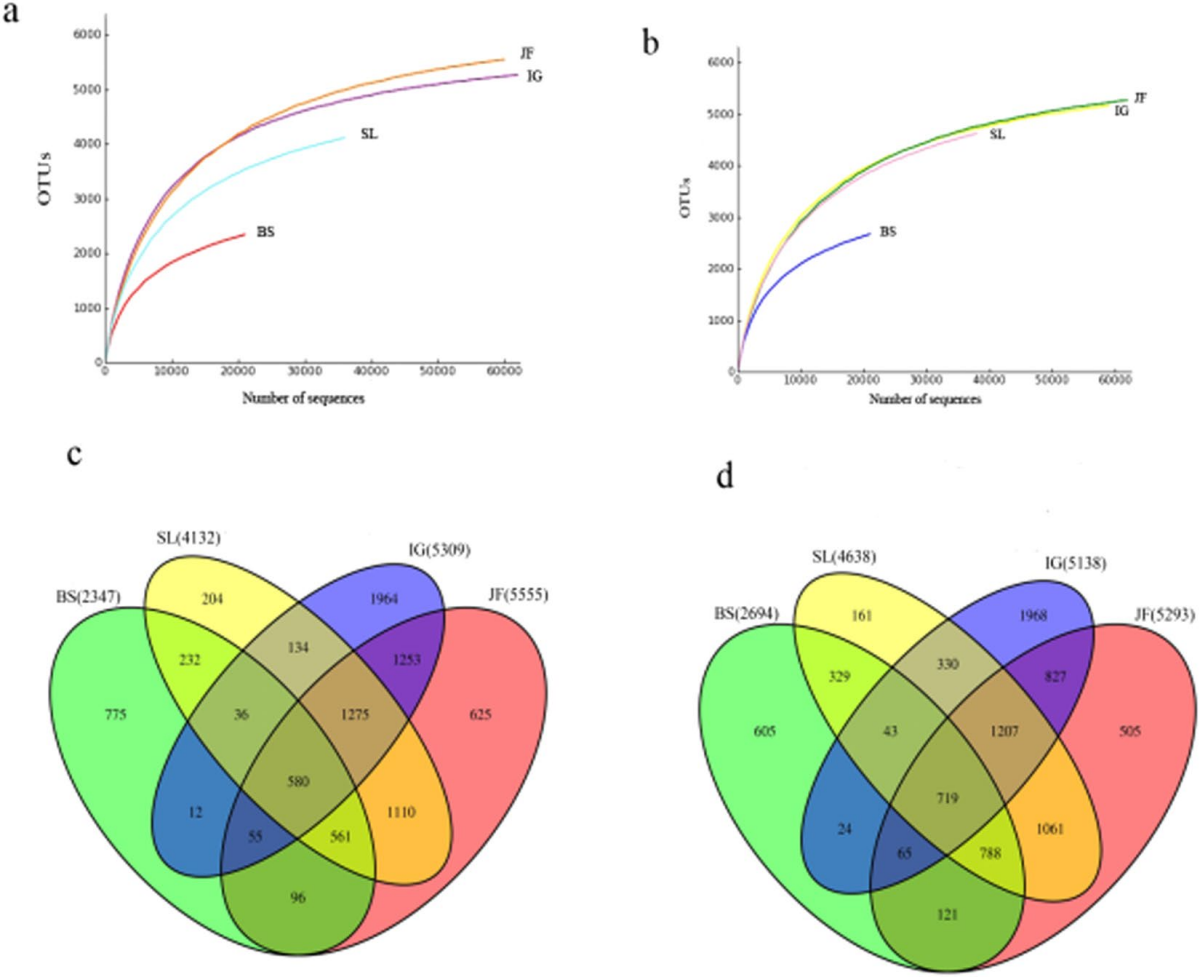
(**a**,**b**) Calculated rarefaction curves of observed OTU richness at four different sites at soil depths 0–10 cm and 10–20 cm. (**c**,**d**) Venn diagram depicting operational taxonomic units (OTUs) detected in soil under different vegetation types at soil depths 0–10 cm and 10–20 cm. Numbers in parentheses indicate the total number of OTUs detected at each site.

The rarefaction curves for BS and SL at 0–10 cm layer and BS at 10–20 cm did not tend toward a plateau, suggesting that the surveying effort did not cover the full extent of taxonomic diversity (Fig. 2a,b). The numbers of OTUs were ranked in the order: JF > IG > SL > BS at either soil depth (Fig. 2c,d).

Bacterial richness (Chao 1) and diversity (Shannon and Simpson) indices were shown in Table 5. The Chao 1 index was highest in JF and lowest in BS at 0–10 cm depth, whereas the ranking of the sites at 10–20 cm depth followed the order JF > IG = SL > BS. The Shannon index at 0–10 cm soil depth showed a decreasing (albeit non-significant) trend from JF to IG to SL, with these three being significantly higher than BS. At 10–20 cm, Shannon index was greater in JF than IG, whereas BS showed the significantly lower Shannon index than the other three sites. Simpson index of diversity showed no appreciable difference among the four sites at the two soil layers (Table 5).

**Table 5:** Comparison of the estimated operational taxonomic unit (OTU) richness and diversity indices of the 16SrRNA gene libraries clustered at 97% identity.

Sites	Soil depth	Chao 1	Shannon index	Simpson diversity
BS	0–10 cm	3085^c^	9.40^b^	0.99^b^
	10–20 cm	3467^b^	9.99^c^	1^a^
SL	0–10 cm	4663^b^	10.53^a^	1^a^
	10–20 cm	5002^a^	10.57^ab^	1^a^
IG	0–10 cm	5039^b^	10.75^a^	1^a^
	10–20 cm	5240^a^	10.19^bc^	1^a^
JF	0–10 cm	5511^a^	11.04^a^	1^a^
	10–20 cm	5158^a^	10.79^a^	0.99^a^

Means (n = 3). Different letters in a column at the same soil layer indicate significant (p ≤ 0.05) differences among the four sites.

**Figure 3:**
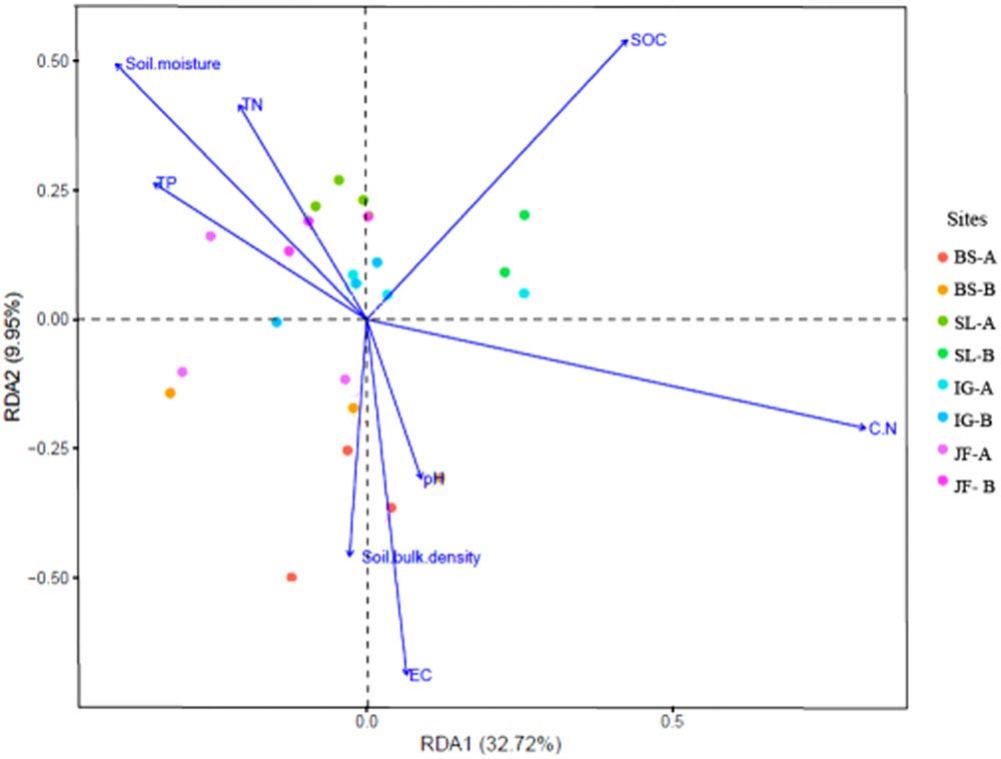
Redundancy discrimination analysis (RDA) depicting the relationship between the main soil physicochemical parameters and diversity distribution of bacterial communities of the four soil sites. Symbols of different colour represent the soil sites/depth and arrows denote physicochemical parameters. The length of each arrow represents the strength of correlation between the environmental factors and community distribution. (**A**) 0–10 cm; (**B**) 10–20 cm depth.

Redundancy discrimination analysis (RDA) was used to analyze the diversity distribution of bacterial community at the four sites in response to soil environmental variables (e.g., TN, TP, SOC, pH, C/N, soil bulk density and EC) (Fig. 3). The first two axes accounted for 32.7% and 10.0% of variation. RDA analysis indicated that SOC and C/N were the most influential factors effecting diversity of bacterial community in soils of the four sites, explaining respectively 97% (P < 0.01) and 61% (P < 0.01) of the total variation.

OTUs and Chao 1, Shannon and Simpson diversity indices each had a significant negative correlation with EC (p < 0.01), but they were positively correlated with SOC, soil moisture, DOC and alkaline phosphatase activity (p < 0.01) (Table S1). MBC had positive correlation with Chao 1 index (0.72, p < 0.01). Urease activity had a positive correlation with OTUs, Shannon index and Simpson diversity. Invertase activity showed a positive correlation with OTUs and Chao 1 index (p < 0.01) (Table S1).

### Composition and relative abundance of bacterial communities

**Figure 4:**
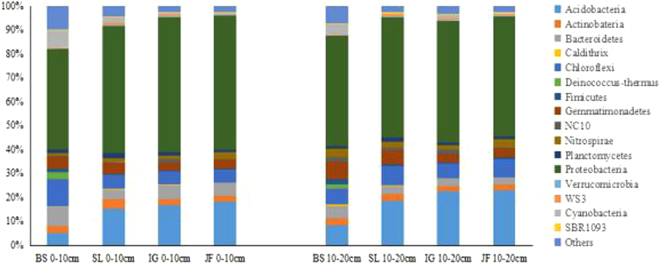
Relative abundance of the dominant bacterial phyla of the four sites at the soil depth 0–10 cm and 10–20 cm. The relative abundances are based on the proportional frequencies of DNA sequences that could be classified at the phylum level.

The relative abundances of the phyla detected are presented in Fig. 4. Soil bacterial community composition included 16 phyla in both soil layers and all four sites. In decreasing order of abundance these 16 phyla were: *Proteobacteria* > *Acidobacteria* > *Chloroflexi* > *Bacteroidetes* > *Gemmatimonadetes* > *Actinobacteria* > *Nitrospirae* > *Planctomycetes* > *Cyanobacteria* > *Fimicutes* > WS3 > NC10 > *Verrucomicrobia* > SBR1093 > *Deinococcus-thermus* > *Caldithrix* > other unidentified phyla. The first eight phyla were the dominant bacterial communities, accounting for 52.8, 18.1, 6.7, 4.6, 4.4, 2.8, 2.3 and 1.4% of total bacteria, respectively. Other phyla each accounted for 0.3–0.7% of the total bacteria community (Fig. 4).

The distribution of each phylum varied among the four sites and with soil depth. *Proteobacteria* was the most abundant phylum in soils following the order SL = IG > JF > BS in the surface 0–10 cm. Compared with BS samples, those from SL, IG and JF had a significantly higher percentage of *Acidobacteria* (respectively, 2.8, 2.9 and 3.3 times in the topsoil 0–10 cm, and also 2.1, 2.6 and 2.7 times in the subsoil 10–20 cm). The relative abundance of *Verrucomicrobia* in the two soil layers followed the order: JF = IG > SL > BS. In the early stage of vegetation succession, the relative abundance of *Actinobacteria* and *Planctomycetes* was higher in SL than BS, but declined in IG. The relative abundance of *Actinobateria* was higher in JF than IG, but the opposite was true for *Planctomycetes*. In contrast, the relative abundances of *Chloroflexi, Bacteroidetes, Fimicutes* and *Cyanobacteria* in the two soil layers were significantly higher in BS than the other three sites (BS > SL > IG > JF).

The relative abundances of 16 phyla differed in the two soil layers. At all four sites, *Verrucomicrobia* and *Deinococcus-thermus* were more abundant in the topsoil than subsoil. In contrast, *Chloroflexi, Gemmatimonadetes, Nitrospirae, Firmicutes*, WS3 and NC10 were relatively more abundant in the subsoil than topsoil.

On a genus level, all 597 detected genera were found in all samples, except for *Gp26*, *Ohtaekwangia*, *Saccharibacteria-genera-incertae-sedis, Streptophyta, Gemmatimonas, Ignavibacterium, Latescibacteria-genera-incertae-sedi, Phenylobacterium, Amaricoccus, Methylibium, Azoarcus, Candidatus-Entotheonella, Plesiocystis, Marinobacterium, Pseudomonas, Arenimonas, Lysobacter, Subdivision3-genera-incertae-sedis, and Steroidobacter* that were not detected in BS, whereas *Aliifodinibius, Gracilimonas, Salinibacter, Dehalogenimonas, GpIX-Truepera, Paenisporosarcina, NP25, Halanaerobium, Rhodoligotrophos, Marivita, Roseovarius, Novispirillum, Rhodovibrio, Desulfatiglans-Desulfococcus, Desulfosalsimonas, Haliea, Methylohalomonas*, and *Halomonas* were detected only in BS. *Gp1, Gp7, Acanthopleuribacter, Aciditerrimonas, Gaiella, Pontibacter, Sediminibacter, Flavitalea, Niastella, Terrimonas, Echinicola, Hyphomonas, Pedomicrobium, Mesorhizobium, Dongia, Piscinibacter, Limnohabitans, Rubrivivax, Geobacter, Kofleria, Pseudoxanthomonas, Opitutus*, and *WPS-2-genera-incertae-sedis* were detected only in JF. *Candidatus, Hydrogenedens*, and *Parcubacteria-genera-incertae-sedis* were detected only in SL*. Vadicella, Azospirillum* and *Thalassobaculum* were detected only in IG.

*Parcubacteria-genera-incertae-sedis, GpXIII, Parvularcula, Loktanella-Erythrobacter, Geoalkalibacter, Marinobacter*, and *Salinisphaera* were detected in BS and SL. *Fulvivirga, Hoeflea, Bauldia, Labrenzia. Desulfopila, Haliangium, Photobacterium*, and *Spartobacteria-genera-incertae-sedis* were detected in SL and IG. *Gp11, Flavobacterium, Devosia, Nitratireductor, Rhodobacter, Porphyrobacter, Kaistobacter-Novosphingobium, Sphingobium, Azohydromonas, Hydrogenophaga, Dechloromonas*, and *Thauera* were detected in IG and JF.

The 27 genera with significant differences among sites and soil depths were listed in Table 6. *GP10, GP15, GP5, GP9* and *Geminicoccus* were more abundant in the subsoil than topsoil at the four sites. In contrast, *GP16* was more abundant in the topsoil than subsoil at the four sites. The abundance of *GP6*, *GP16*, *Rhodoplanes, Steroidobacter* and *Sphingomonas* in the topsoil (0–10 cm) followed the order: JF > IG > SL > BS.

**Table 6:** The genera showing significant differences in relative abundance (%) in the microbial community at the four sites differing in vegetation types.

Taxon	Soil depth	Site			
		BS	SL	IG	JF
*GP10*	0–10 cm	0.57 ± 0.19b	1.4 ± 0.15a	1.47 ± 0.19a	1.25 ± 0.05a
	10–20 cm	1.15 ± 0.05b	2.40 ± 0.38a	1.63 ± 0.33ab	1.37 ± 0.03b
*GP15*	0–10 cm	0.1 ± 0.01b	0.57 ± 0.07a	0.45 ± 0.05a	0.50 ± 0.01a
	10–20 cm	0.17 ± 0.07b	0.80 ± 0.10a	0.7 ± 0.01a	0.6 ± 0.06a
*GP17*	0–10 cm	0.25 ± 0.05c	0.90 ± 0.10a	0.65 ± 0.15ab	0.5 ± 0.06bc
	10–20 cm	0.65 ± 0.05b	0.85 ± 0.05ab	0.70 ± 0.20b	1.55 ± 0.35a
*GP2*	0–10 cm	0.10 ± 0.01b	0.53 ± 0.07a	0.53 ± 0.19a	0.35 ± 0.05ab
	10–20 cm	0.15 ± 0.05b	0.70 ± 0.06a	0.35 ± 0.05ab	0.70 ± 0.01a
*GP21*	0–10 cm	1.80 ± 0.78ab	3.33 ± 0.83a	2.85 ± 0.45ab	0.7 ± 0.26b
	10–20 cm	7.90 ± 1.90a	4.37 ± 0.76b	6.60 ± 0.30ab	1.30 ± 0.25c
*GP22*	0–10 cm	0.20 ± 0.01b	0.37 ± 0.03a	0.23 ± 0.03b	0.20 ± 0.06b
	10–20 cm	0.35 ± 0.05a	0.37 ± 0.03a	0.30 ± 0.06a	0.20 ± 0.06a
*GP25*	0–10 cm	0.1 ± 0.01a	0.20 ± 0.06a	0.20 ± 0.06a	0.30 ± 0.10a
	10–20 cm	0.50 ± 0.01a	0.20 ± 0.06a	0.15 ± 0.05a	0.50 ± 0.21a
*GP3*	0–10 cm	0.37 ± 0.12b	1.17 ± 0.18b	0.97 ± 0.12b	2.30 ± 0.46a
	10–20 cm	0.6 ± 0.12b	0.90 ± 0.06b	0.73 ± 0.07b	1.63 ± 0.20a
*GP5*	0–10 cm	0.13 ± 0.03b	1.33 ± 0.28a	0.73 ± 0.19b	0.57 ± 0.12b
	10–20 cm	0.20 ± 0.01b	1.83 ± 0.41a	1.13 ± 0.24ab	1.10 ± 0.17ab
*GP6*	0–10 cm	0.07 ± 0.01c	2.43 ± 0.43b	2.93 ± 0.56ab	4.33 ± 0.54a
	10–20 cm	0.33 ± 0.23c	2.33 ± 0.44b	2.07 ± 0.62b	6.10 ± 0.45a
*GP9*	0–10 cm	0.23 ± 0.08b	0.83 ± 0.03a	0.67 ± 0.09ab	0.37 ± 0.09b
	10–20 cm	0.33 ± 0.12b	0.93 ± 0.09a	0.80 ± 0.07a	1.00 ± 0.06a
*GP16*	0–10 cm	0.10 ± 0.01b	0.57 ± 0.09a	0.63 ± 0.24a	0.67 ± 0.03a
	10–20 cm	0.15 ± 0.05c	0.60 ± 0.01b	0.70 ± 0.01ab	0.85 ± 0.05a
*GP18*	0–10 cm	0.10 ± 0.01a	0.10 ± 0.01a	0.10 ± 0.01a	0.10 ± 0.01a
	10–20 cm	0.10 ± 0.01a	0.10 ± 0.01a	0.10 ± 0.01a	0.10 ± 0.01a
*Ilumatobacter*	0–10 cm	0.10 ± 0.01b	0.63 ± 0.09a	0.25 ± 0.05b	0.27 ± 0.03b
	10–20 cm	0.10 ± 0.01a	0.23 ± 0.09a	0.23 ± 0.13a	0.13 ± 0.03a
*Salisaeta*	0–10 cm	0.20 ± 0.01a	0.13 ± 0.03ab	0.10 ± 0.01b	0.03 ± 0.01ab
	10–20 cm	1.05 ± 0.95a	0.17 ± 0.03a	0.10 ± 0.01a	0.10 ± 0.01a
*Nitrospira*	0–10 cm	0.85 ± 0.15b	1.03 ± 0.09b	0.63 ± 0.03b	1.83 ± 0.17a
	10–20 cm	1.60 ± 0.76a	1.10 ± 0.06a	0.97 ± 0.03a	1.73 ± 0.03a
*Geminicoccus*	0–10 cm	4.27 ± 0.49a	3.93 ± 0.45a	3.63 ± 0.28ab	2.5 ± 0.42b
	10–20 cm	7.90 ± 0.50a	4.63 ± 0.39b	4.15 ± 0.05b	4.40 ± 0.20b
*Hyphomicrobium*	0–10 cm	0.1 ± 0.01a	0.13 ± 0.03a	0.17 ± 0.03a	0.17 ± 0.03a
	10–20 cm	0.15 ± 0.05a	0.10 ± 0.01a	0.13 ± 0.03a	0.17 ± 0.03a
*Rhodoplanes*	0–10 cm	0.10 ± 0.01b	0.20 ± 0.06b	0.27 ± 0.03b	0.50 ± 0.08a
	10–20 cm	0.40 ± 0.01a	0.17 ± 0.07b	0.17 ± 0.03b	0.37 ± 0.03a
*Afifella*	0–10 cm	0.17 ± 0.03b	0.33 ± 0.03a	0.37 ± 0.07a	0.17 ± 0.03b
	10–20 cm	0.20 ± 0.06b	0.57 ± 0.03a	0.60 ± 0.02a	0.17 ± 0.03b
*Pelagibius*	0–10 cm	0.25 ± 0.05b	1.27 ± 0.20a	0.97 ± 0.18a	0.77 ± 0.12ab
	10–20 cm	0.20 ± 0.01b	0.83 ± 0.09a	1.00 ± 0.15a	0.63 ± 0.13ab
*Desulfuromonas*	0–10 cm	0.13 ± 0.0.3c	0.67 ± 0.03a	0.43 ± 0.03b	0.23 ± 0.09c
	10–20 cm	0.35 ± 0.05a	0.30 ± 0.10a	0.33 ± 0.07a	0.30 ± 0.06a
*Thioprofundum*	0–10 cm	0.20 ± 0.06b	0.23 ± 0.03ab	0.47 ± 0.12a	0.27 ± 0.03ab
	10–20 cm	0.33 ± 0.03a	0.27 ± 0.07a	0.25 ± 0.05a	0.27 ± 0.03a
*Steroidobacter*	0–10 cm	0.10 ± 0.07b	0.47 ± 0.09b	1.40 ± 0.17a	1.83 ± 0.26a
	10–20 cm	1.10 ± 0.01b	0.67 ± 0.09c	1.00 ± 0.15b	1.60 ± 0.01a
*Sphingomonas*	0–10 cm	0.10 ± 0.01b	0.20 ± 0.06b	0.23 ± 0.03b	1.33 ± 0.35a
	10–20 cm	0.10 ± 0.01b	0.10 ± 0.02b	0.13 ± 0.03b	0.37 ± 0.12a

Means ± standard error (n = 3); means followed by different letters in a row (one-way ANOVA) are significantly different at P ≤ 0.05.

## Discussion

The soil properties are influenced by parent materials, topography, climate, and organisms. Soil moisture, depth, structure, fertility, pH, and salinity are the main factors that influence plant community distribution and species composition^33^. In the study reported here, the effects of vegetation succession in coastal saline-alkali land on soil physical and chemical properties were obvious. EC and soil bulk density decreased with the vegetation succession. In contrast, SOC and TN increased (Table 1). This indicated that vegetation succession could improve the physical and chemical properties of coastal saline-alkali soil.

SOC is the largest terrestrial organic carbon pool and plays a vital role in the global C cycle^34^. SOC is mainly derived from litter, root and plant residues and root exudates and is affected by species composition in the vegetation, land use and management^35^. Our study showed that SOC content was significantly lower in BS than in SL, IG and JF. The largest increase in SOC occurred between BS and SL, and smaller increases were recorded with the vegetation succession (Table 2), which was consistent with related studies^36^.

MBC represents an important living part of soil organic matter, and is involved in the processes influencing soil development^37^. Any changes in soil MBC have a significant impact on soil carbon, nitrogen, phosphorus, plant species and the dynamics of the terrestrial ecosystems^38^. It can be used as the early prediction index of soil quality and the change of soil total organic matter^39^. Our results showed that there was an increasing trend of soil MBC with the succession of vegetation community (Table 3). This may be explained by an increase in soil fertility and increased rhizodeposition through vegetation succession^40^.

DOC is composed of organic compounds mainly derived from root exudates, microbial biomass and the decomposition of plant litter and soil organic matter^41^. DOC is the most active form of soil organic carbon and plays an important role in the global carbon cycling^42^. In this study, DOC concentrations showed an increasing trend with the succession of vegetation communities (Table 3). DOC had positive correlation with OTUs and the Shannon, Simpson diversity and Chao 1 indices (Table S1), suggesting that the process of vegetation succession results in an increase in root exudates and other easily soluble carbon components, which enhances microbial growth and diversity.

Soil EC was significantly negatively correlated with SOC content, total P, urease and invertase activities, but positively correlated with soil bulk density (Table 4). These findings suggested that a decrease in soil salt content was associated with the succession of vegetation communities, resulting in an increase in SOC content and the improvement in soil fertility and soil structure.

Soil enzyme activity reflects the rate of soil nutrient cycling and utilization and may be an index of soil biodiversity, productivity and potential microbial activity^43^. Soil enzymes are linked with vegetation community variations^44^. In the present study, activities of soil urease, catalase, invertase and alkaline phosphatase at both soil depths were highest in JF, followed by IG and SL, with the lowest values measured in BS, suggesting vegetation succession enhanced soil nutrient transformation (Fig. 1). Soil EC was negatively correlated with activities of most soil enzyme in our study. In contrast, SOC was positively correlated with urease and invertase activity, whereas TP was positively correlated with catalase and invertase activity, suggesting that SOC and soil nutrient content had a substantial effect on soil enzyme activities. Alkaline phosphatase activity was correlated with urease activity, and that of catalase to invertase activity (Table 4), reflecting general inter-dependence of at least some soil enzymes in promoting the soil nutrient cycling.

Soil microbial communities play a central role by driving soil organic matter decomposition and nutrient cycling^45^. Bacteria are the most abundant and diverse group of microorganisms, playing an important role in maintaining soil health and ecological services^46^. The increases in the Chao 1 and Shannon indices were ranked generally in the order: JF > IG > SL > BS (Table 5), indicating that the richness and diversity of soil bacterial communities increased with the succession of vegetation in the coastal saline-alkali soil.

Diversity of microbial communities was controlled by biotic and abiotic factors^47^. Soil pH, SOC, C/N ratio, moisture, temperature, and soil types were the major determinants of the diversity of soil microbial community^48^. The RDA analysis showed that the diversity of soil bacteria in various stages of vegetation succession was related mainly to C/N and SOC (Fig. 3). The species richness (Chao 1) and diversity (Shannon and Simpson) indices had a significant positive correlation with SOC and DOC (p < 0.01) (Table S1), indicating that with the succession of plant communities (accompanied by an increase in SOC and DOC and a decrease in EC) soil became a more suitable environment for supporting abundant and diverse microbial communities to enhance the ecosystem stability in coastal saline-alkali land. However, other factors affecting distribution and abundance of bacteria cannot be excluded.

The 16S rRNA gene sequencing results indicated an increasing diversity of bacteria with vegetation community succession in coastal saline-alkali soil. *Proteobacteria, Acidobacteria, Chloroflexi, Bacteroidetes, Gemmatimonadetes, Actinobacteria, Nitrospirae* and *Planctomycetes* were the dominant bacterial phyla across the whole succession sequence. Carbon storage can be increased through N deposition, N_2_ fixation and N fertilizer supply to the ecosystem^49^. *Proteobacteria* are one of the largest phyla of soil bacteria and include many nitrogen-fixing bacteria^50^. JF had the highest percentage of *Proteobacteria*, followed by IG and SL, with the lowest in BS.

Correlation analysis among soil properties and microbial diversity parameters indicated that OTUs and the Shannon, Chao 1 and Simpson diversity indices had a negative correlation with EC, but a positive correlation with soil moisture and SOC (Table S1). These results indicated that vegetation succession of coastal saline-alkali soil associated with decreased soil salinity might have resulted in increased abundance and diversity of bacterial communities, including those bacterial phyla with relatively low resistance to high soil salinity.

The percentages of *Deinococcus-thermus, Fimicutes* and *Cyanobacteria* showed a decreased trend, whereas the percentages of *Acidobacteria* and *Proteobacteria* revealed an increasing trend in the process of vegetation succession (Fig. 4). *Cyanobacteria* are widely distributed in freshwater, marine and terrestrial ecosystems, even in the most extreme niches^51,52^. In the present study, compared with SL, IG and JF at 0–10 cm soil depth, BS had a significantly higher percentage of *Cyanobacteria* (3.3, 11.4 and 26.7 times, respectively), indicating relatively extreme and unfavorable conditions in highly saline soil at the BS (bare soil) site. *Acidobacteria* are ubiquitous and play an important role in soil ecological processes^53^. Related research showed that abundance of *Acidobacteria* displayed a more significant positive correlation with soil carbon content than with soil pH^54^. In our study, the percentages of *Acidobacteria* increased with the succession of vegetation communities (Fig. 4), mimicking the trends in SOC (Tables 1 and 2) and DOC (Table 3).

The relative abundances of phyla exhibited an inconsistent response to soil layers. *Verrucomicrobia* are ubiquitous in soil but less frequent bacterial phyla^55^. Relative abundance of *Verrucomicrobia* was highest in the subsurface soils due to their oligotrophic strategies, which was consistent with related research^56^. More research is required to determine whether this phylum has some specific relationships with soil carbon content.

*Salinibacter* is a genus of extremely halophilic red bacteria that are among the most salt-tolerant and salt-requiring strains^57,58^. Similarly, *Paenisporosarcina* is a well-known genus in saline and hypersaline environments and *Desulfosalsimonas* is isolated from the extreme hypersaline sediments^59^. These genera were found in the present study only in BS due to hypersaline conditions. Some uncultured organisms such as *Candidatus* were found in SL with the succession of vegetation. *Azospirillum* was only detected in IG, representing the best-characterized genus of plant growth-promoting rhizobacteria that was found in the rhizosphere of several grasses^60^. *Aciditerrimonas, Pontibacter* and *Flavisolibacte*r that are obligate aerobic genera^61–63^ were detected only in JF. These findings indicated that the process of vegetation succession resulted in differences in soil microbial functional diversity that would have influenced ecosystem nutrient fluxes and soil organic matter quality to a large extent^64^.

## Conclusions

Vegetation succession was associated with the changes in the soil physicochemical properties in coastal saline-alkali soil (eg. decreased soil EC and soil bulk density, and increased SOC and TN) (Fig. S1). Active components of SOC such as DOC and MBC showed an increasing trend with vegetation succession. The SOC and DOC had positive correlations with OTUs and the Shannon, Simpson diversity and Chao 1 indices, suggesting an environment that would enhance bacterial growth and diversity. Activities of soil urease, catalase, invertase and alkaline phosphatase generally increased with vegetation succession. The SOC, TP and soil EC had significant effects on soil enzyme activity. Soil enzyme activity correlated with the species richness and diversity indices, reflecting the level of soil microbial activity. The changes in bacteria richness and diversity were associated with vegetation types, soil moisture, EC, SOC and DOC. Vegetation community succession in coastal saline-alkali soil was associated with increased bacterial diversity. Bacteria such as *Proteobacteria* (that include many nitrogen-fixing bacteria) and *Acidobacteria* (with a significant positive correlation with soil carbon content) may have contributed to promoting the storage of soil organic carbon.

## Electronic supplementary material

Table S1

Fig. S1
