# Supplementary material for: Vegetation succession influences soil carbon sequestration in coastal alkali-saline soils in southeast China
Source: Sci Rep. 2018 Jun 27;8:9728. doi: 10.1038/s41598-018-28054-0 (PMC6021427; doi:10.1038/s41598-018-28054-0)
Supplement: Supplementary file 1 — Table S1 [file 41598_2018_28054_MOESM1_ESM.pdf]

Vegetation succession influences soil carbon sequestration in coastal alkali-saline soils in southeast China

Niu Li[a], Tianyun Shao[a], Tingshuo Zhu[a], Xiaohua Long[a*], Xiumei Gao[a], Zhaopu Liu[a], Hongbo Shao[b], Zed Rengel[c]

[a] *College of Resources and Environmental Sciences, Nanjing Agricultural University, Nanjing 210095, China*

[b] *Salt-soil Agricultural Center, Institute of Agricultural Resources and Environment, Jiangsu Academy of Agricultural Sciences, Zhongling Street 50, Nanjing 210014, China*

[c] *Soil Science and Plant Nutrition, School of Earth and Environment, The University of Western Australia, 35 Stirling Highway, Crawley WA 6009, Australia*

[*] Correspondence: Xiaohua Long, College of Resources and Environmental Sciences, Nanjing Agricultural University, Nanjing 210095, P.R. China. E-mail: longxiaohua@njau.edu.cn

Table S1 Correlation coefficients between soil physicochemical properties or soil enzyme activities and richness and diversity of bacterial communities.

	OTUs	Shannon index	Chao 1	Simpson diversity
pH	-0.45	-0.34	-0.68**	-0.23
EC	-0.86**	-0.86**	-0.82**	-0.76**
Soil moisture	0.82**	0.79**	0.89**	0.73**
SOC	0.73**	0.79**	0.74**	0.73**
MBC	0.48	0.42	0.72**	0.40
DOC	0.74**	0.71**	0.85**	0.67*
Urease	0.70*	0.77**	0.54	0.72**
Catalase	0.09	0.06	0.36	0.13
Alkaline phosphatase	0.80**	0.72**	0.63**	0.65**
Invertase	0.58**	0.56	0.67**	0.54

EC, soil electrical conductivity; SOC, soil organic carbon; MBC, microbial biomass carbon; DOC, dissolved organic carbon. **Significant at $P < 0.01$; *significant at $P < 0.05$.
