# Supplementary material for: Vegetation succession influences soil carbon sequestration in coastal alkali-saline soils in southeast China
Source: Sci Rep. 2018 Jun 27;8:9728. doi: 10.1038/s41598-018-28054-0 (PMC6021427; doi:10.1038/s41598-018-28054-0)
Supplement: Supplementary file 2 — Fig. S1 [file 41598_2018_28054_MOESM2_ESM.pdf]

Vegetation succession influences soil carbon sequestration in coastal alkali-saline soils in southeast China

Niu Li[a], Tianyun Shao[a], Tingshuo Zhu[a], Xiaohua Long[a*], Xiumei Gao[a], Zhaopu Liu[a], Hongbo Shao[b], Zed Rengel[c]

[a] *College of Resources and Environmental Sciences, Nanjing Agricultural University, Nanjing 210095, China*

[b] *Salt-soil Agricultural Center, Institute of Agricultural Resources and Environment, Jiangsu Academy of Agricultural Sciences, Zhongling Street 50, Nanjing 210014, China*

[c]*Soil Science and Plant Nutrition, School of Earth and Environment, The University of Western Australia, 35 Stirling Highway, Crawley WA 6009, Australia*

[*] Correspondence: Xiaohua Long, College of Resources and Environmental Sciences, Nanjing Agricultural University, Nanjing 210095, P.R. China. E-mail: longxiaohua@njau.edu.cn

Supplementary figure legends

Fig. S1 The influence of the interaction of vegetation succession, selected soil physico-chemical properties, soil enzymes, and soil microbe abundance and diversity on soil carbon sequestration in coastal saline-alkali soil.

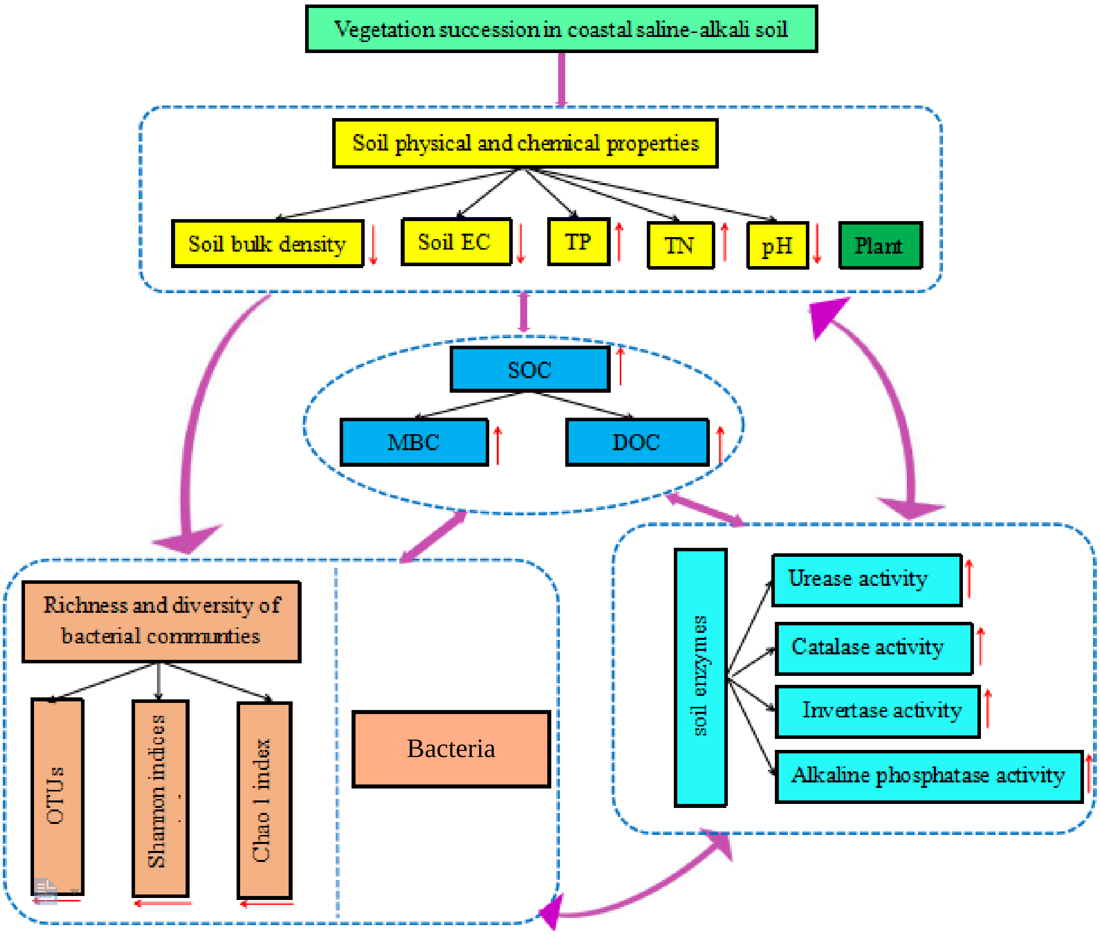

Figure S1: The influence of the interactions among vegetation succession, selected soil physico-chemical properties [electrical conductivity (EC), total nitrogen (TN), total phosphorus (TP)] (yellow), soil enzymes (cyan), and soil microbial abundance and diversity (light brown) on soil carbon sequestration [soil organ carbon (SOC), soil microbial biomass carbon (MBC) and dissolved organic carbon (DOC)] (blue) in coastal saline-alkali soil. Red arrows indicate an increase (up) or a decrease (down or left) with the vegetation succession.
